# Are Kynurenines Accomplices or Principal Villains in Dementia? Maintenance of Kynurenine Metabolism

**DOI:** 10.3390/molecules25030564

**Published:** 2020-01-28

**Authors:** Masaru Tanaka, Zsuzsanna Bohár, László Vécsei

**Affiliations:** 1 MTA-SZTE, Neuroscience Research Group, Semmelweis u. 6, H-6725 Szeged, Hungary; 2 Department of Neurology, Interdisciplinary Excellence Centre, Faculty of Medicine, University of Szeged, Semmelweis u. 6, H-6725 Szeged, Hungary

**Keywords:** dementia, Alzheimer’s disease, kynurenines, kynurenic acid, neuroprotective agents, antioxidant molecules, multitarget agents

## Abstract

Worldwide, 50 million people suffer from dementia, a group of symptoms affecting cognitive and social functions, progressing severely enough to interfere with daily life. Alzheimer’s disease (AD) accounts for most of the dementia cases. Pathological and clinical findings have led to proposing several hypotheses of AD pathogenesis, finding a presence of positive feedback loops and additionally observing the disturbance of a branch of tryptophan metabolism, the kynurenine (KYN) pathway. Either causative or resultant of dementia, elevated levels of neurotoxic KYN metabolites are observed, potentially upregulating multiple feedback loops of AD pathogenesis. Memantine is an N-methyl-D-aspartate glutamatergic receptor (NMDAR) antagonist, which belongs to one of only two classes of medications approved for clinical use, but other NMDAR modulators have been explored so far in vain. An endogenous KYN pathway metabolite, kynurenic acid (KYNA), likewise inhibits the excitotoxic NMDAR. Besides its anti-excitotoxicity, KYNA is a multitarget compound that triggers anti-inflammatory and antioxidant activities. Modifying the KYNA level is a potential multitarget strategy to normalize the disturbed KYN pathway and thus to alleviate juxtaposing AD pathogeneses. In this review, the maintenance of KYN metabolism by modifying the level of KYNA is proposed and discussed in search for a novel lead compound against the progression of dementia.

## 1. Introduction

Dementia, currently known as major neurocognitive disorder (NCD) in the Diagnostic and Statistical Manual of Mental Disorders Fifth Edition (DSM-5), is an acquired cognitive decline of six discrete cognitive domains including complex attention, executive function, learning and memory, language, perceptual-motor function, and/or social cognition. Alzheimer’s disease (AD) is the most common form of NCDs accounting for 60% to 70%, while other etiological causes include frontotemporal neurocognitive disorder, Lewy bodies, vascular cognitive disorder (VCD), traumatic brain injury, substance or medication, HIV infection, prion disease, Parkinson’s disease (PD), Huntington’s disease (HD), another medical condition, or multiple etiologies [1]. About 50 million people suffer from dementia in the world, and there are nearly 10 million new cases every year. Five to 8% of the population aged more than 60 years old suffers from dementia. The total number of patients with dementia is estimated to 82 million in 2030 and 152 million in 2050, and much of the increase is attributed to low- and middle-income countries. Dementia causes not only dependency and disability among the elderly, but it also imposes a physical, psychological, social, and economic burden to people with dementia as well as their families, caregivers, and society [2].

Initially identified by Alois Alzheimer in 1906 and later named by Emil Kraepelin in 1910, AD is an irreversible chronic neurodegenerative disease beginning with the gradual onset of memory loss, mood disturbance, or changes in language or thinking skills and progressing to disturbance of personality and behaviors. The electron microscopic discovery of neurofibrillary tangles (NFTs) in brain biopsies was followed by findings of other positive lesions including amyloid beta (Aβ), neuropil threads, and dystrophic neurites containing hyperphosphorylated tau accompanied by astrogliosis. Negative lesions include losses of neurons, neuropils, and synaptic elements, which are largely associated with tangle formation [3]. The neurodegenerative lesions of postmortem brain samples of AD patients correlate well with imaging studies. Shrinkage of the hippocampus in the early stage and the significant shrinkage of many brain regions in the later stage are shown by structural imaging studies including magnetic resonance imaging (MRI) and computerized tomography (CT) [4]. Regional patterns of the brain shrinkage may help identify affected cognitive domains and diagnose other causes of dementia. Low uptake and the reduced level of glucose in the cognitive domains of the brain in the early stage can be revealed by functional brain imaging such as positron emission tomography (PET) and functional MRI (fMRI) [5].

The pathological and clinical discoveries have led to propose several hypotheses of AD pathogenesis and thus, much effort has been devoted to design drugs painstakingly to target at etiological entities such as Aβ, tau proteins, neurotransmitter receptors, etc. In the meantime, some AD hypotheses have been realized to align in a pathological sequence to merge as a series of harmful cellular and neural events in a cascade and furthermore, to potentiate the pathological consequence in a vicious cycle by the presence of positive feedback loops.

This review article presents an alignment of proposed hypotheses in the cascade of AD, the presence of positive feedback loops, and a systematic review on the status of bioactive kynurenines (KYNs) in major NCD to support the concept of KYNs as participants of new positive feedback loops in AD. In association with N-methyl-D-aspartate (NMDA) receptors (NMDARs) modulator memantine, which is an AD drug so far approved for clinical use, a multitarget kynurenic acid (KYNA) is discussed and proposed as a novel lead compound for the maintenance of KYN metabolism, which potentially leads to alleviate the vicious pathological cycles of AD.

A literature search was employed in PubMed/MEDLINE and Google Scholar, using appropriate search terms and filters according to a theme of each section, and a systematic review was conducted to synthesize studies of human samples regarding the status of KYNs in neurodegenerative diseases and psychiatric disorders that affect cognitive domains, as described in detail in Appendix A.

## 2. Convergence of Alzheimer’s Disease Pathogenesis

Neurodegenerative atrophy of the brain in dementia has been associated with amyloid plaques and NFTs derived from hyperphosphorylated tau in AD pathogenesis [6,7]. The pathological landmarks of AD were also observed in age-related mitochondrial dysfunction, proposing a mitochondrial cascade hypothesis that mitochondrial dysfunction activates downstream cellular events including Aβ amyloidosis, neuroinflammation, oxidative stress, tau phosphorylation, synaptic loss, and finally neurodegeneration in late-onset AD [8]. More comprehensive AD pathophysiology advocates the inflammation hypothesis. Injury elicits a recruitment of leukocytes to the site of lesion and a subsequent activation of the brain microglia and astrocytes, resulting in neuroinflammation [9]. Biomarkers associated with both the innate and adaptive immune system are increased in blood, serum, plasma, and cerebrospinal fluid (CSF) of AD patients. Unresolved and prolonged reactions lead to a disruption of pro-inflammatory and anti-inflammatory cytokine balance, causing chronic inflammation. Pro-inflammatory interleukin (IL)-1β, IL-6, tumor necrosis factor (TNF)-α and anti-inflammatory cytokines, IL-1 receptor antagonist, and IL-10 are elevated both in the plasma and CSF of AD patients [10]. It is worth noting that the levels of anti-inflammatory cytokines are elevated in AD, PD, and HD, but they are reduced in VCD [11,12,13,14,15]. Numerous evidences suggest that in addition to neuroinflammation, dementia is associated with systemic inflammation, which is responsible for a risk factor, component, and progression of dementia [16]. Furthermore, an imbalance in the gastrointestinal microbiota has been described to induce inflammation that is associated with neurodegenerative disorders such as AD and PD [17].

**Figure 1:**
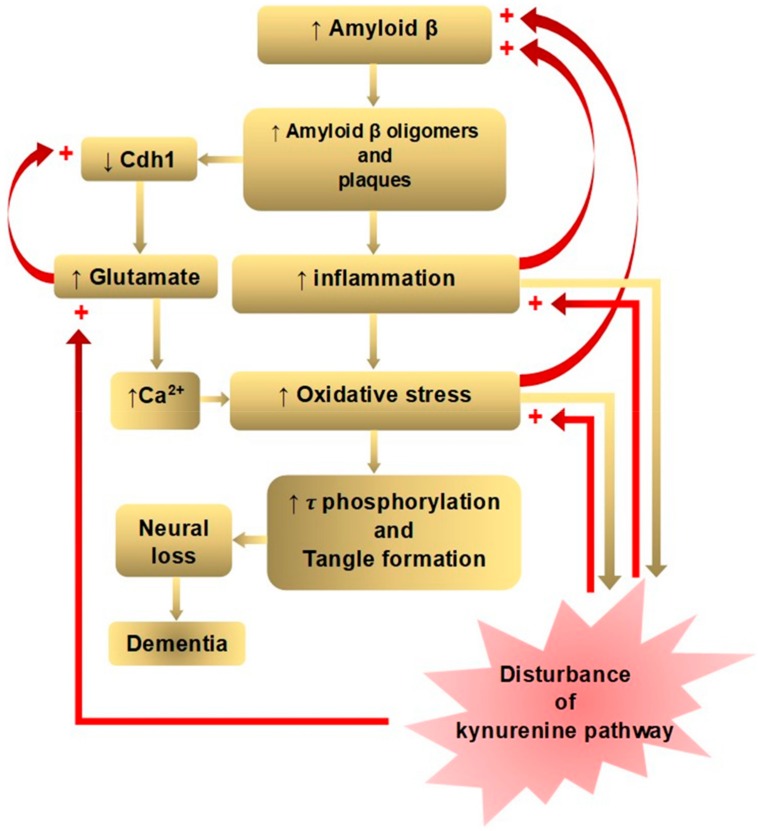
Positive feedback loops of amyloid β hypothesis of Alzheimer’s disease in connection with disturbance of the kynurenine pathway. The amyloid beta (Aβ) cascade, inflammation, tau phosphorylation, and neuroplasticity hypotheses lie in one downward cascade, and the glutamate and calcium hypotheses lie in another branched downward cascade of pathological events leading to dementia. Positive feedback loops are located between inflammation and increased Aβ accumulation, between increased oxidative stress and increased Aβ accumulation, and increased glutamate and decreased cadherin 1 CDh1). Kynurenine (KYN) pathway enzymes are activated by inflammation: the tryptophan dioxygenase (TDO) by the glucocorticoid stress hormone, cortisol and indolamine-2,3-dioxygenase (IDO1) by pro-inflammatory cytokines, interferon (IFN)-α, interleukin (IL)-1β, IFN-γ, and tumor necrosis factor (TNF)-α. IFN-γ also activates formamidase and kynurenine-3-monooxygenase (KMO) in human microglia and macrophages. KYN pathway metabolites, 3-hydroxykynurenine (3-HK) and quinolinic acid (QUIN) are highly reactive free radicals. In addition, QUIN is an *N*-methyl-d-aspartate receptor (NMDAR) agonist, causing excitotoxicity. Thus, disturbance of the KYN pathway potentiates inflammation, oxidative free radical attack, and excitotoxic glutamate production (partly adopted from Doig, 2018).

A dominant model of AD, the amyloid cascade hypothesis, holds that an abnormal accumulation of Aβ plaques in the interneurons of the brain triggers a cascade of events: microglia and astrocytes activation, chronic inflammation, increased glutamate levels, elevated intracellular calcium, oxidative stress, synaptic dysfunction, tangle formation by tau hyperphosphorylation, neuronal loss, and finally dementia [18]. The Aβ accumulation and deposition in the interneurons lead to microglial activation, cytokine release, reactive astrocytosis, and an induction of inflammation [19]. Aβ oligomers also cause the proteasome-dependent degradation of cadherin 1 (Cdh1), which is responsible for downregulation of glutaminase: an enzyme that converts glutamine to glutamate [20]. Glutaminase has been found to be elevated in the prefrontal cortex of AD patients [21]. Glutamate also decreases Cdh1 to inactivate antigen-presenting cell (APC)/C-Cdh1, leading to a further accumulation of glutaminase, creating a positive feedback loop [20]. Increased glutamate causes a sustained low-level activation at the glutamate receptors, including NMDARs. This chronic excitotoxic insult leads to neuronal death and cognitive impairment, which has been proposed by glutamate hypothesis [22]. The elevated glutamate level increases the intraneuronal Ca^2+^ level, which is another pathway leading to neuronal apoptosis [23]. Aβ oligomers can directly trigger Ca^2+^ flux through the plasma membrane, increasing intracellular Ca^2+^ concentration. Ca^2+^ signaling plays important roles in cellular function as a second messenger, involving entry and release channels, clearance mechanisms, and intracellular stores [24]. Increased Ca^2+^ levels can also lead to mitochondrial Ca^2+^ overload, superoxide radicals-induced oxidative stress, and pro-apoptotic mitochondrial proteins production, as proposed in calcium homeostasis hypothesis [25] (Figure 1).

The activation of excitatory glutamatergic neurotransmission is critical for synaptic plasticity. The synaptic NMDAR activation initiates plasticity, but the activation of extrasynaptic NMDAR impairs neuroplasticity and results in cell death [26]. Neuroplasticity is the dynamic morphological and functional changes of remodeling the synapses, axons, and dendrons including neurogenesis and synaptogenesis, forming new connections, pathways, and circuits. The process accounts for memory, learning, cognitive functions, and compensation initiated by injury and disease [27]. A higher level of neuroplasticity was observed in the hippocampus, neocortical areas, and cholinergic basal forebrain responsible for the regulation of higher brain functions [28]. The brain regions with elevated neuronal plasticity are the most vulnerable in aging and in AD, as proposed by neuroplasticity hypotheses [29]. A disproportion between synapse formation and elimination can be responsible for defective plasticity during aging and neurodegenerative disease. Defective mechanisms controlling the plasticity may contribute to inefficient plasticity processes [27]. Memory deficits in AD could be related to early events that come before neurodegeneration, such as synaptic loss and dysfunction. A cholinergic hypothesis was proposed by both anatomical findings of selective cholinergic neuron loss in the basal forebrain and clinical improvement in AD patients treated with acetylcholine (Ach) esterase inhibitors [30]. A deficient neurotrophic hypothesis was proposed by the selective loss of cholinergic neurons and the discovery of neurotrophic factors such as nerve growth factor [31].

The exacerbation of oxidative stress leads to abnormally increased phosphorylated tau proteins polymerizing to form NFTs. Tau proteins are microtubule-associated proteins, which play an important role in the assembly of microtubules and stability of microtubules network in neurons. The dysfunction of tau proteins affects the structural and regulatory functions of the cytoskeleton, leading to abnormal axonal transport, synaptic dysfunction, impaired neuroplasticity, and neurodegeneration [32]. A tau knockout mouse (tau^−/−^ mice) study showed that the absence of tau leads to a decrease in functional extrasynaptic NMDARs in the hippocampus, and it was proposed that tau deficiency causes the extrasynaptic NMDAR impairment contributing to neuroprotective effects [33].

AD hypotheses have been proposed according to anatomical, clinical, and medicinal findings, but a single hypothesis fails to elucidate AD pathogenesis. Numerous failed clinical trials have led to assume a presence of multiple heterogenous etiologies of AD genotypes and phenotypes, eventually converging to a common pathological and clinical vignette. Neurodegeneration can be reached along different pathways in AD subtypes [34]. Nevertheless, each hypothesis is closely connected, and many positive feedback loops exist to exacerbate the disease process. The amyloid cascade, inflammation, tau phosphorylation, and neuroplasticity hypotheses lie in one downward cascade, and the glutamate and calcium hypotheses lie in another branched cascade of pathological events, leading to dementia. One positive feedback loop is located between inflammation and increased Aβ accumulation, and the other loop bridges increased oxidative stress and increased Aβ accumulation. Another positive feedback loop occurs between increased glutamate and decreased Cdh1, leading to a further accumulation of glutaminase. The presence of multiple positive feedback loops may contribute to exacerbate the pathological consequences in AD [35] (Figure 1).

## 3. Multiple Positive Feedback Loops via Kynurenine Metabolites

Dementia patients have been associated with the disturbance of tryptophan (TRP) metabolism and its downward catabolic branch, the KYN pathway. Low circulating TRP levels, elevated neurotoxic KYN metabolites, and a reduced neuroprotective KYN metabolite are observed in elderly patients with neurodegenerative disease such as AD, PD, and HD [36]. Either causative or resultants of AD pathogenesis, the aberrant KYN pathway lies not only in a close connection with AD pathophysiology but also may play a critical role in potentiating the multiple positive feedback loops of AD pathology.

The KYN pathway transforms over 95% of TRP into a series of small bioactive molecules with neurotoxic, neuroprotective, oxidative, or antioxidative properties. Inflammation activates several key enzymes in the pathway [37]. The indole ring of TRP is oxidized to produce N-formyl KYN by the TRP dioxygenase (TDO) in the liver, the indolamine-2,3-dioxygenase (IDO) 1 in the brain, and peripheral tissues and IDO 2 in the liver, kidney, and antigen-presenting cells [38]. TDO is activated by the glucocorticoid stress hormone, cortisol; IDO1 is activated by the pro-inflammatory cytokines, interferon (IFN)-α, IL-1β, IFN-γ, and TNF-α, and it is inhibited by the anti-inflammatory cytokines, IL-2, IL-4, IL-10, and transforming growth factor-β (TGF-β) through IFN-γ. IDO2 knockout mouse (IDO2^−/−^ mice) revealed that IDO2 has a pro-inflammatory role and contributes to autoantibody production [39]. Thus, stressful events and inflammatory responses activate the rate-limiting TRP enzymes to cascade down in the KYN pathway.

N-formyl KYN is converted by formamidase to L-KYN, which is a substrate of three downstream metabolites: anthranilic acid (AA) by kynureninase, 3-hydroxy-KYN (3-HK) by KYN-3-monooxygenase (KMO), and KYNA by pyridoxal 5′-phosphate (PLP)-dependent KYN aminotransferases (KATs) [40]. AA and its metabolite, 3-hydroxy-AA (3-HAA), are found to suppress pro-inflammatory cytokine IFN-γ, T and B lymphocyte cell proliferation, and Th1 cell activity and invoke anti-inflammatory cytokine, IL-10 [41]. 3-HK generates highly reactive free radicals. An elevation of 3-HK levels has been shown to be related to excitotoxic injury and is observed in patients with neurodegenerative diseases [42].

A KAT isoform, KAT II, functions in the physiological pH range and may be responsible for most of the KYNA synthesis in the brain. KATs also convert 3-HK to xanthurenic acid (XA) [43]. KYNA is an antagonist at ionotropic α-amino-3-hydroxy-5-methyl-4-isoxazolepropionic acid (AMPA), NMDA, kainate glutamate receptors, and the α7 nicotinic Ach receptor [44]. However, the role of KYNA at the α7 nicotinic Ach receptor remains controversial [45]. KYNA binds to the G protein-coupled receptor (GPR) 35 (GPR35) expressed in glia, macrophages, and monocytes to reduce glutamate release in brain and pro-inflammatory cytokine release in cell lines. KYNA also binds to aryl hydrocarbon receptor (AhR) to alleviate adaptive immune responses [46].

**Figure 2:**
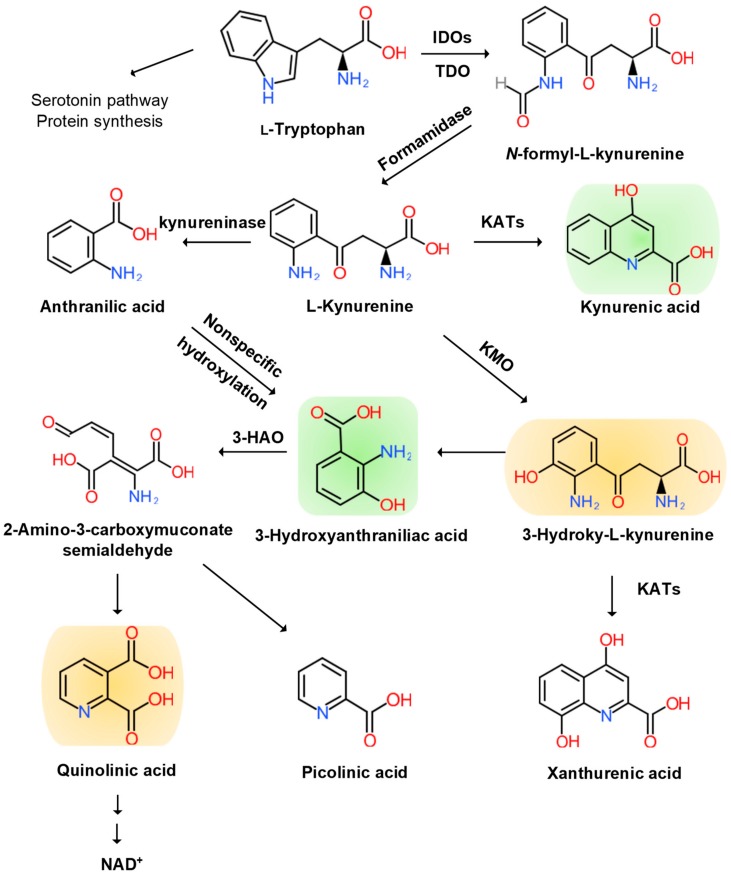
Kynurenine Branch of Tryptophan Metabolism. More than 95% of tryptophan is metabolized in the kynurenine (KYN) pathway except for serotonin metabolism and protein synthesis. Tryptophan (TRP) is converted to KYN by the hepatic rate-limiting tryptophan 2,3-dioxygenase (TDO) and ubiquitous rate-limiting indoleamine 2, 3-oxygenase (IDO) 1, each of which is induced by cortisol, and interferon (IFN)-α, IFN-γ, and tumor necrosis factor (TNF)-α, respectively. KYN is converted to anthranilic acid (AA) by the kynureninase, 3-hydroxy-L-kynurenine (3-HK) is converted by the KYN-3-monooxygenase (KMO), and kynurenic acid (KYNA) is converted by KYN aminotransferases (KATs). KYNA is an antagonist at the NMDA receptor. AA and 3-HK are converted to 3-hydroxyanthranillic acid (3-HAA) and further to picolinic acid (PIC) and quinolinic acid (QUIN). 3-HK and QUIN are agonists at the NMDA receptor. QUIN is converted to nicotinamide adenine dinucleotide (NAD^+^), which is a feedback inhibitor of TDO. Neurotoxic KYNs are shown in orange, and neuromodulartory KYNs are shown in green.

3-HK and AA are converted by 3-hydroxyanthranilate oxidase to highly redox-active 3-HAA, which may play a role in the regulation of oxidative stress. 3-HAA suppresses cytokine and chemokine production and neurotoxicity induced by IL-1 or IFN-γ [47]. 3-HAA is converted by 3-hydroxyanthranilate dioxygenase to 2-amino-3-carboxymuconate semialdehyde, which is further transformed into picolinic acid (PIC) and an excitotoxic and free-radical metabolite, quinolinic acid (QUIN). The pro-inflammatory cytokine IFN-γ stimulates IDO, formamidase, and kynurenine-3-monooxygenase (KMO) activities in human microglia and macrophages, leading to increased QUIN synthesis. The activation of macrophages and glial cells induces the increased production of QUIN [48]. Anti-inflammatory steroid agents such as dexamethasone suppress QUIN concentrations in the brain following immune stimulation [49]. Finally, QUIN is metabolized in subsequent steps into nicotinic acid dinucleotide (NADH) (Figure 2).

## 4. Systematic Review on Kynurenines in Major Neurocognitive Disorders

A systematic review was conducted on the status of KYNs in major NCD. Inclusion criteria, exclusion criteria, selection process, data extraction, assessment of the methodological quality, and the risk of bias assessment are described in Appendix A. A total of 30,004 articles matched our database search. Out of 586 articles 10 meta-analysis and systematic reviews, a total of 212 articles were assessed for eligibility. Finally, 23 articles were deemed for synthesis in this systematic review. The methodological quality and risk of bias assessment are shown in Table 1. Evidence levels of neurotoxic and neuromodulatory KYN levels were assessed at low risk of bias for MDD; high risk of bias for AD, PD, and HD; unclear of bias for VCD, bipolar disorder (BP), generalized anxiety disorder (GAD), and autism spectrum disorders (ASD) (Table 1).

### 4.1. Kynurenines in Neuodegenerative Diseases

Increased KYN, KYNA, and QUIN in serum and CSF were associated with aging [50]. Altered levels of KYN metabolites have been observed in patients with AD, PD, HD, and VCD. An increased KYN/TRP ratio of the plasma and CSF, increased levels of IDO in the brain, and immunoreactivity for both IDO and QUIN in the microglia, astrocytes, and neurons of hippocampal tissue were observed in AD [51,52,53,54]. It has been suggested that KYNs are involved in the regulation of glutamate neurotransmission, neuroprotection, and immune responses in AD. Furthermore, an increased CSF 3-HK/KYN ratio was correlated with t-tau and p-tau, while plasma KYN and PIC inversely correlated with p-tau and t-tau, respectively [55]. KYNA levels are decreased in the plasma, witnessing the shift toward neurotoxic metabolites over neuroprotective ones in AD [53]. Higher and lower levels of KYN were associated with a higher Neuropsychiatric Inventory (NPI) total score, and a lower KYN/KYNA ratio indicated risk for hallucination in AD and Lewy bodies dementia [56].

The plasma samples of PD patients showed significant lower activities of KAT I and KAT II with a decreasing tendency of plasma KYNA levels [57]. A metabolomic profiling study of CSF from PD patients showed increased 3-HK levels [58]. A metabolomic evaluation showed that a lower KYNA/KYN ratio, higher QUIN level, and higher QUIN/KYNA ratio were observed in the plasma of PD patients, suggesting a shift toward neurotoxic QUIN and away from neuroprotective KYNA synthesis [59]. The alterations in KYN metabolite levels may contribute to pathogenesis in PD, and the KYN pathway intervention was proposed to alleviate PD symptoms through neuroprotection [60].

The KYN/TRP ratio was higher, while the KYNA/KYN ratio was lower in the plasma of HD patients than controls [61]. A postmortem brain study showed decreased KYN levels in the middle and inferior cortex, decreased KYNA levels in the precentral gyrus, frontal, and temporal cortex, and decreased 3-HK levels in the inferior temporal cortex [62]. Another study also showed decreased KYNA levels in the caudate nucleus and lower KAT I and KAT II in the putamen of HD patients [63]. However, 3-HK levels were significantly higher in the frontal and temporal cortex in HD brain samples [64]. A significant reduction in TRP levels was found at several days after stroke onset, and the KYN/TRP ratio was elevated much higher in stroke patients [65]. KYNA levels were higher in patients who died within 21 days after stroke [66].

Many studies have presented disturbance of KYN metabolism in patients with dementia. Increased levels of neurotoxic KYNs were observed in AD, PD, HD, and VCD. It is intriguing that levels of neuroprotective KYNA were decreased in AD, PD, and HD, but increased in VCD. Further study is expected to uncover the status and change of neurotoxic and neuroprotective KYN metabolites under progression of the diseases (Table 2).

### 4.2. Kynurenines in Psychiatric Disorders

Cognitive domains are also affected in psychiatric disorders such as major depressive disorder (MDD), bipolar disorder (BD), generalized anxiety disorder (GAD), and autism spectrum disorders (ASD). Lower levels of plasma TRP, KYN, and KYNA were observed in MDD. A higher level of QUIN immunoreactivity was detected in the prefrontal cortex and hippocampus of the postmortem samples of MDD patients [67,68,69]. Chronic stress has been linked in MDD to structural brain damages including a loss of dendritic spines and synapses, reduced dendritic arborization, and diminished glial cells in the hippocampus [70]. A possible relationship between KYN metabolism and suicide ideation has been investigated in psychiatric patients, including non-MDD patients. Higher levels of CSF QUIN, a higher ratio of CSF QUIN/KYNA, and lower levels of CSF KYNA have been associated with suicide attempts in psychiatric patients [71]. Lower levels of PIC, lower ratio of PIC/QUIN, and a higher ratio of KYN/TRP were reported in patients with suicide attempts. However, studies have not reached a consensus on the upregulation or downregulation of TDO/IDO enzymes among the suicide-prone population [72].

Cognitive deficits of verbal/visual memory and executive tasks have been observed during depressive episodes in BD, while executive dysfunction and attention deficits have been reported during manic episodes in BD [73,74]. A case-control study reported increased 3-HK/KYN and 3-HK/KYNA ratio and decreased KYNA levels in BD [75]. A meta-analysis reported an increased level of KYNA in the CSF of bipolar patients [76]. However, another meta-analysis reported no significant difference of TRP and KYN levels, nor KYN/TRP and KYNA/QUIN ratios in serum from BD patients [77]. Further intensive study is expected on the status of the KYN metabolites in manic and depressive phases of BD patients. In patients with GAD, decreased levels of plasma KYN were observed in endogenous anxiety and normalized after treatment [78]. Significantly lower levels of KYN have been associated with Type D personality, which has been characterized by negative affectivity and social inhibition [79].

The status of KYN metabolites has not reached a consensus in ASD. The blood KYN and QUIN levels and KYN/TRP ratio were found significantly higher, PIC levels were significantly lower, and KYNA levels were unchanged in ASD [80]. The serum KYNA level was significantly lower, while the KYN/KYNA ratio was significantly higher in children with ASD [81]. The results have not reached consensus, which is most probably due to a small number of studies and the heterogenous etiologies of ASD (Table 2).

**Figure 3:**
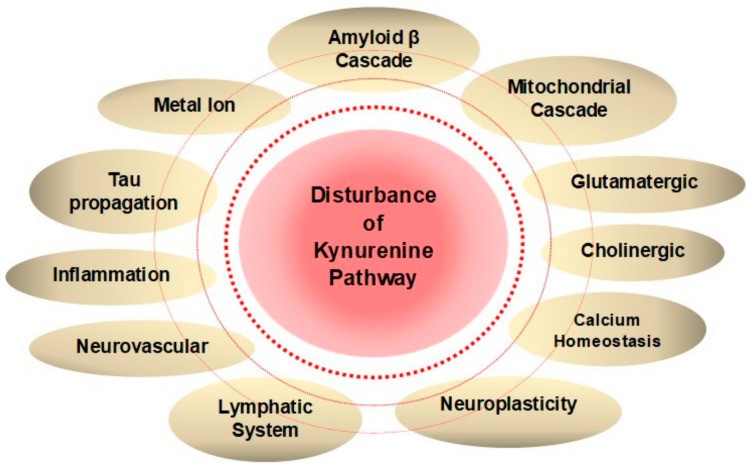
Disturbance of Kynurenine Metabolism Wires Multiple Positive Feedback Loops of Alzheimer’s Disease. Hypotheses of Alzheimer’s disease (AD) pathogenesis derived from anatomical, clinical, and medicinal findings are closely connected to each other, and many positive feedback loops exist to exacerbate the disease. Disturbance of a branch of tryptophan metabolism, kynurenine (KYN) pathway lies in a close connection with various pathogeneses of dementia. Increased neurotoxic KYN metabolites and decreased neuroprotective kynurenic acid (KYNA) may potentiate multiple feedback loops of AD pathogenesis.

It is intriguing that lower levels of KYNA is associated with psychiatric disorders affecting cognitive domains, but higher levels of KYNA is observed in patients suffering from schizophrenia, which barely exhibits cognitive symptoms [76,82]. Further investigation is expected on the relationship between KYN metabolism and psychiatric disorders. Disturbance of TRP and KYN metabolisms has been observed in patients suffering from major NCD and is found to be closely linked to AD pathogenesis and dementia in which multiple positive feedback loops through an imbalance of KYN metabolites may potentially contribute to the exacerbation of dementia (Figure 3).

## 5. Tolerogenic Shift of Adaptive Immune Response by Kynurenine Metabolites

Besides excitotoxic, inflammatory and oxidative insults, disturbance of KYN metabolism directs the adaptive immune response to tolerogenic status. Activation of the KYN pathway suppresses effector T cells and induces regulatory T cells (Tregs), leading immune status to a tolerogenic state [83]. Upon IDO activation by stress and inflammatory response, TRP depletion activates the stress response kinase, general control non-depressible 2 (GCN2) by binding to uncharged tRNA. GCN2 activation leads to downregulation of the CD3 zeta (ζ)-chain in CD8^+^ T cells, blockage of T helper (Th) 17 cell (Th17) cell differentiation and cell cycle entry by T cell receptor-activated T cells, and activation of resting CD4^+^ Tregs [84]. TRP depletion also inhibits the nutrient-sensing mammalian target of rapamycin 1 pathway to inhibit T effector cell function and growth [85].

IDO-activated cells can transform the function of APCs producing pro-inflammatory cytokine, IL-12, into anti-inflammatory cytokines including TGF-β and IL−10 [86]. IDO increases the level of KYN, which mediates the inhibition of IL-2 signaling to reduce CD4 T-cell survival [87]. Binding to AhR, KYN induces the dendritic cell and macrophage differentiation, which initially induces a highly inflammatory CD4^+^ T-cell subset, Th17 cells, and then further differentiate into Tregs during the resolution of inflammation [88]. In addition, KYNA and XA are endogenous human AhR ligands. KYN, KYNA, and XA direct the adaptive immunity toward immune suppression [89].

IDO-expressing cells promote the differentiation of CD4^+^ T cells into Treg cells expressing CTLA-4, which is a protein receptor that functions as an immune checkpoint and downregulates immune responses [90]. In addition, higher KYNA production and lower KMO expression are associated with another regulatory immune mechanism, contributing to dysfunctional effector CD4^+^ T-cell response [87]. NAD^+^ induces the apoptosis of naïve CD4^+^ T-cells and reduces the number of Tregs, but it protects differentiated Th1, Th2, Th17, from CD4^+^ T-cells and induced Treg against apoptosis [91]. Thus, KYN metabolites and enzymes generally convert local immunogenic T cell functions to tolerogenic ones.

Meanwhile, a population-based cohort study showed that doubling of the derived granulocyte-to-lymphocyte ratio, platelet-to-lymphocyte ratio, and systemic immune–inflammation index were associated with an increased dementia risk, suggesting an imbalance in the immune system and dominance of the innate over adaptive immune system in the pathogenesis of dementia [92]. Thus, a dominant innate immune response with a tolerogenic shift of adaptive immune response may help perpetuate chronic inflammation.

## 6. NMDA Receptor Modulator, Memantine

New drug candidates under clinical trials are targeting Aβ, cholinergic neurotransmission, NMDARs, tau proteins, neurovasculation, inflammation, or virus [93]. Memantine belongs to the NMDAR modulators, which is one of only two classes of medications so far approved for the treatment of AD [94]. Thus, it is worth exploring new lead compounds among NMDAR antagonists.

Initially synthesized and patented in 1968 for the treatment of diabetics, an adamantane derivative, memantine, failed to lower blood sugar levels, but it was found to improve the cognitive performance of severely demented patients in a Phase III human clinical trial in 1999. Memantine is now approved to use for the treatment of moderate to severe AD, and combination therapy with cholinesterase inhibitors offers better outcomes including cognitive and behavioral symptoms [95].

Anti-NMDAR property had been discovered in 1980s. The NMDAR is essential for processes such as learning and memory. An excessive activation of NMDAR was shown to be associated with neuronal damage and loss contributing to various acute and chronic neurological disorders, including dementia. Nevertheless, physiological NMDAR activity is essential for normal neuronal function, and any agents that block all NMDAR activity have unacceptable clinical side effects [96]. The glutamatergic receptor modulators are under intensive study for the development of novel drugs against mood disorders such as BP [97].

NMDARs are tetramers consisting of two GluN1 and two GluN2 or GluN3 subunits. Four subtypes of GluN2 (A–D) and two subtypes of GluN3 (A–B) are identified. The subunits composition constantly changes during development and according to neural activity, determining the distinct biophysical, pharmacological and signaling properties of NMDAR. For example, tri-heteromeric GluN1/GluN2A/GluN2B receptors are responsible for long-term potentiation induction at adult CA3–CA1 synapses [98]. Pathologic overstimulation of the NMDAR causes a chronically active state initiating excitotoxicity and has been implicated in neurodegenerative diseases such as strokes, AD, HD, and amyotrophic lateral sclerosis [98].

Memantine, a noncompetitive, low-affinity, voltage-dependent antagonist of NMDARs preferentially enters the receptor-associated ion channel to prevent calcium current flow when it is excessively open but does not interfere with normal synaptic transmission. Thus, it prevents or protects against further damage from neuronal cell death induced by excitotoxicity. The fast on-and-off neurotransmission and low–moderate affinity are the keys to memantine action because it blocks the effects of excessive glutamate while preserving the physiologic activation of NMDARs required for learning and memory [99].

Memantine was reported to inhibit the extrasynaptic NMDAR more effectively than synaptic NMDAR [100]. Furthermore, the preferential NMDAR inhibition of the memantine subpopulation has been studied. The NMDAR subunit GluN2A abundant in the synaptic NMDAR, mediate the neuroprotective pathway, while the GluN2B subunit, which is abundant in the extrasynaptic NMDAR, mediates the neurotoxic pathway. It was also proposed that a higher mobility of the NMDAR subunit GluN2B-containing NMDAR enhances availability in the extrasynaptic sites than less mobile GluN2A-containing NMDAR [101]. However, the distribution of subunits is not strictly limited to the synaptic or extrasynaptic sites. It was also proposed that an increased occupancy of GluN1-2A by memantine induces NMDAR desensitization by intracellular Ca^2+^ accumulation, contributing to the inhibition of NMDAR subpopulations. Thus, memantine inhibition depends upon Ca^2+^ concentration, NMDAR subtype, and the intensity of NMDAR activation [102]. However, little is known about the exact mechanism of memantine to alleviate AD symptoms, and thus further investigation is expected.

Memantine is also an antagonist at the nicotinic Ach and serotonergic (5-HT) type 3 (5-HT_3_) receptors. A majority excreted unchanged in urine (75%–90%), but three polar metabolites, the N-gludantan conjugate, 6-hydroxy memantine, and 1-nitroso-deaminated memantine, possess minimal NMDAR antagonist activity [100,103].

Other NMDAR antagonists and modulators have been investigated and entered clinical trials. Gavestinel (GV150,526A), an NMDAR antagonist that binds selectively to the glycine site on the NMDAR complex, was found to be a potent neuroprotective agent in animal models of stroke such as permanent middle cerebral artery occlusion in the rat. It reached Phase III clinical trials; however, it was concluded to show no efficacy in treating ischemic stroke [104]. AVP-786 (trade name Nuedexta), a combination drug of a weak NMDAR antagonist dextromethorphan hydrobromide and quinidine sulfate, enhances its calming effect. It is approved by the FDA for the treatment of pseudobulbar affect and is under clinical trial for the treatment of agitation in patients with dementia of AD. However, it was reported to have no benefit in three Phase 3 trials against the agitation of AD [105]. AXS-05 is a combination drug of bupropion (a norepinephrine–dopamine reuptake inhibitor and nicotinic Ach receptor antagonist) and dextromethorphan (a sigma-1 receptor agonist, NMDAR antagonist, and serotonin–norepinephrine reuptake inhibitor) for the treatment of treatment-resistant MDD and agitation in AD [106]. BI425809 is a potent and selective glycine transporter 1 (GlyT-1) inhibitor that modulates the level of glycine, a co-agonist of NMDAR, for the treatment of cognitive impairment of AD and schizophrenia [107]. DAOI is an NMDAR modulator under Phase 2 clinical trials, which is hypothesized to have better efficacy than the placebo for cognitive function in patients with AD [108] (Table 3).

Either with a combination of other bioactive compounds, the new drug candidates under clinical trials possess a broad range of biological activities besides NMDAR modulation. Rational drug design better focuses on multitarget strategy in addition to specific etiological targets of dementia to tune the nervous activities properly. Furthermore, the NMDAR target strategy may well benefit from focusing on compounds with modulatory NMDAR properties. Memantine is not strictly a NMDA antagonist. It is a NMDAR modulator with weak agonistic activity and multiple target sites including 5-HT_3_ receptor and nicotinic Ach receptor activities. A search for multitarget molecules may be of great value to discover possible lead compounds against dementia.

## 7. Maintenance of Kynurenine Metabolism to Alleviate Multiple Positive Feedback Loops

Modifying a level of KYNA to balance a disturbed KYN pathway may help alleviate the multiple positive loops of AD pathogenesis. Patients with AD, PD, HD, and MDD has been found to have decreased levels of KYNA, which has multiple targets and actions including anti-excitotoxic, anti-inflammatory, antioxidant, and immunomodulatory activities.

Firstly, the neuroprotective effects of KYNA are attributed to the inhibition of glutamate excitotoxicity. KYNA binds to the strychnine-insensitive glycine-binding site of the NR1 subunit at lower concentration (EC_50_ = 7.9 to 15 μM), while at higher concentrations, it blocks the glutamate-binding site to the NR2 subunit of NMDAR (EC_50_ = 200 to 500 μM) [109,110]. KYNA was reported to inhibit the presynaptic α7 nicotinic Ach receptors (IC_50_ = ~7 μM), but it has not been confirmed by an in vivo study. More evidences support the view that KYNA may not influence nicotinic Ach receptors [45,111]. Furthermore, KYNA exhibits a dual effect at AMPA receptors in a dose-dependent manner: KYNA inhibits at micromolar concentrations, but at nanomolar concentrations, it evokes facilitation through allosteric modulation of the AMPA receptor [112].

Secondly, KYNA has been observed to have anti-inflammatory actions under inflammatory conditions. KYNA reduces TNF expression and secretion, diminishes high-mobility group box 1 protein secretion, inhibits α-defensin human neutrophil peptides 1–3 secretion, reduces IL-4 release in invariant natural killer-like T cells, reduces lipopolysaccharide-induced IL23 expression of dendritic cells, and inhibits Th17 cell differentiation in vitro [89].

Thirdly, KYNA has been shown to activate GPR35 signaling, through which it reduces glutamate release in the brain, reduces pro-inflammatory cytokines release in the glia and macrophages, and exerts the analgesic effects in inflammatory models. GPR35 activation induces N-type calcium channel inhibition, which contributes to the regulation of neuronal excitability and synaptic transmitter release [113]. In addition, GPR modulators are an emerging class of novel drugs under clinical trials against various diseases including diabetics, cardiovascular diseases, and psychiatric disorders such as depression, bipolar disorder, and schizophrenia [114].

Fourthly, another target of KYNA is a xenobiotic receptor, the AhR, which plays roles in the regulation of cellular differentiation, cellular adhesion and migration, and immune response. AhR controls adaptive immunity by modulating T-cell differentiation and function directly and indirectly [115]. The expression of IDO is sustained by an autocrine loop in the presence of AhR and KYNA in tumor infiltrating tolerogenic DCs and a positive feedback loop controlled by AhR drives IL-6 expression, and it sustains IDO expression and KYN production in tumor cells. AhR activation by KYNA is considered an important regulator of immunotolerance via the IL-6-dependent pathway in tumors. Furthermore, LPS-induced immune response was limited by AhR [116].

Fifthly, KYNA is an antioxidant that possesses reactive oxygen species (ROS) scavenging activities observed in various in vitro models and can prevent tissue damage triggered by overshooting inflammation. KYNA (100 μM) can abolish ROS production produced by FeSO_4_, which is a molecule with a mechanism of toxicity primarily involving O^2−^ and OH production [117]. Decreased levels of KYNA may provoke an inadequate anti-inflammatory response, resulting in enhanced tissue damage and exceeding cell proliferation during inflammatory in AD, PD, and HD [41]. Increased production of KYNA may be compensatory to limit the inflammatory reaction in AD.

Finally, an increased KYNA level stimulates the kynureninase A, which converts KYN to AA, resulting in an elevation of AA [118]. AA may possess potential anti-inflammatory properties either by itself or via its 5-hydroxylated metabolites. AA is metabolized to 3-HAA by a microsomal hydroxylase in mammalian liver. An expected anti-inflammatory reaction AA is derived from the fact that AA is a precursor of some nonsteroidal anti-inflammatory drugs such as mefenamic acid and diclofenac [119]. AA suppresses pro-inflammatory IFN-γ, T and B lymphocyte cell proliferation, and Th1 cell activity, while it increases anti-inflammatory cytokine IL-10 [120]. In addition, the KYNA level may be influenced by the substrate availability, KAT enzyme activity, and its degradation rate. The induction of other branches of TRP metabolism may also be relevant for the synthesis of serotonin and melatonin, both of which are also immune regulators (Table 4).

## 8. Kynurenic Acid-Targeted Approaches: Strategies, Alternatives, and Considerations

The blood–brain barrier (BBB) is poorly permeable to KYNA. The design of KYNA precursors that are highly penetrable across the BBB and convertible to an active form upon the entry has been under consideration. Another strategy is the administration of KYNA analogues that are highly penetrable to the BBB. The halogenation and conjugation of various side chains enables KYNA to cross the BBB easily, and the KYNA analogues have been shown to be more potent NMDAR inhibitors. Meanwhile, inadequate nutritional status has been observed in patients with dementia. An active form of vitamin B_6_, PLP is a cofactor of KAT enzymes, which are responsible for KYNA production. Therefore, vitamin B_6_ supplementation may be of important value to increase a level of KYNA in the brain. L-KYN is not only a precursor of KYNA, which is also produced at least partly from indole pyruvic acid (IPA) through redox reactions or the transamination of TRP. Little is studied about other routes of KYNA production and its influence on whole KYN metabolism. In addition, D-enantiomers of amino acids and D-amino acid oxidase (DAAO) have been observed to contribute to L-amino acid concentration. D-TRP and D-KYN supplements and balancing the gastrointestinal microbiota responsible for conversion to L-enantiomers may be potential strategies to regulate KYN metabolism and maintain an adequate L-KYNA reservoir.

### 8.1. Prodrugs

The peripheral administration of KYNA precursor, KYN was found to lead to neuroprotection in hypoxic-ischemic animal models [121]. The peripheral administration of 4-chloro-KYN or 4,6-dichloro-KYN leads to the formation of 7-chloro-KYNA or 5,7-dichloro-KYNA in the brain and more potent antagonists at the glycine site of NMDARs than KYNA [122]. An orally active L-4-Cl-KYN known as AV-101 showed efficacy in animal models of HD and neuropathic pain [46,123]. However, a Phase II clinical trial had shown negative results against MDD in 2019 [124]. The development of other BBB-penetrating prodrugs is expected to be explored.

### 8.2. Kynurenic Acid Analogues

More potent NMDAR-modulating KYNA derivatives have been synthesized in the search for promising new neuroprotective agents [125]. Halogenated KYNA analogues presented significantly lower IC50 values than the parent compound, and chlorination in position 7 of KYNA increased the affinity for the Gly site of the NMDARs [126,127]. Fluorination in position 5 and chlorination in position 5, 7, or 5 and 7 increased potency in the antagonism of glutamate-induced ileal contraction and for [^3^H]Gly binding assay [128]. Hippocampal and entorhinal cortical applications of 7-Cl-KYNA attenuated magnesium-induced seizures in vitro. Intrahippocampal 5,7-di-Cl-KYNA injection prevented the behavioral and the electrographic manifestations in a rat model of status epilepticus [129]. The microinfusion of 5,7-di-Cl-KYNA suppressed the effect of both glutamate- and glycine-induced seizures of freely moving rats [130]. Bilateral 5,7-di-Cl-KYNA injection into the rostral striatum inhibited the haloperidol-induced muscle rigidity in rats, which is an animal model of parkinsonian-like muscle rigidity [131]. 4-trans-2-carboxy-5,7-dichloro-4-phenylaminocarbonylamino-1,2,3,4-tetrahydroquinoline, 5,7-di-Cl-KYNA, and 7-Cl-KYNA showed neuroprotective effects against glutamate-induced excitotoxicity in rat cortical neurons [132]. Thiokynurenates are also potent non-competitive antagonists of the NMDARs. Substitution of a thio group for the hydroxyl group in position 4 of KYNA increased the potency and chlorination of position 7 or 5 and 7 of 4-thio-KYNA and further increased potency in ileal myenteric plexus and for [^3^H]Gly binding [133]. 4-urea-5,7-di-Cl-KYNA derivatives exerted anticonvulsant activity in maximal electroshock, subcutaneous pentylenetetrazole, and threshold tonic extension tests in mice [133].

BBB-penetrating KYNA derivatives have been synthesized by esterization. The methyl ester of diphenylureido-di-Cl-KYNA appeared to be protective against audiogenic seizures in DBA/2 mice [134]. d-Glucose or d-galactose esters of 7-Cl-KYNA penetrate the BBB and are converted to 7-Cl-KYNA or KYNA by astrocytes and neurons in the brain. d-Glucose esters of 7-Cl-KYNA and d-galactose esters of 7-Cl-KYNA attenuated the NMDA-induced seizures probably by increasing the BBB penetration [135]. The intraventricular and intravenous administration of glucose-KYNA induced stereotyped behaviors and ataxia and transient reductions of the amplitude of the somatosensory-evoked cortical potentials, suggesting that glucose-KYNA possesses similar activities to KYNA and crosses the BBB [136]. A KYNA amide derivative, *N*-(2-*N*,*N*-dimethylaminoethyl)-4-oxo-1*H*-quinoline-2-carboxamide hydrochloride showed electrophysiological properties similar to KYNA in vitro and showed a neuroprotective effect in models of cerebral ischemia (four-vessel occlusion) and an HD model of transgenic mice [47,137].

Nanotechnology-based approaches are under intensive study to overcome the blood–brain barrier and deliver the appropriate amount of drug to the specific brain site. Organic nanocarriers include polymeric nanoparticles, liposomes, dendrimers, and micelles, while inorganic nanocarriers include gold nanoparticles, silica nanoparticles, and carbon nanotubes [138]. Further research is expected to understand the blood–brain barrier crossing mechanisms and to improve the efficiency of brain delivery methods using nanotechnology.

### 8.3. KAT Enzyme Potentiation

KYN metabolism can be shifted toward KYNA production by enhancing KAT enzyme activity. KATs catalyzes the irreversible transamination of KYN to produce KYNA. The enzyme requires a cofactor, PLP, the active form of vitamin B_6_, and a cosubstrate, α-ketoacid. The kinetics of KATs depends on local KYN availability ascribed to its low affinity for their substrate. The active form of vitamin B_6_, PLP, is a cofactor in many enzymes [139]. A main source of PLP is food and degraded PLP-dependent enzymes by salvage pathway enzymes in humans. Genetic dysfunction of the salvage pathway enzymes and drug interactions of PLP or pyridoxal kinase results in convulsions and epileptic encephalopathy, and a lower level of PLP has been associated with neurological disorders including AD, PD, and epilepsy [140,141]. About 20% of the elderly have been observed to have lower dietary vitamin B_6_ intakes and other nutrients, and a daily intake of 20 mg vitamin B_6_ improves vitamin B_6_ status in healthy older men and vitamin B_6_ supplementation improves cognitive performance in elderly men. It has been hypothesized that folate and vitamins B_6_ and B_12_ are related to cognitive performance [142,143]. Vitamin B_6_ emerged as a good predictor of cognitive performance across cognitive domains, but whether B_6_ supplementation can improve cognitive performance is still to be demonstrated through ongoing longitudinal clinical trials. A correlation between blood levels of B vitamins and cognitive function has been documented, and high vitamin B_6_ concentration has been correlated with better performance in memorization tests [144].

Nutrition status is relevant to the onset of dementia. Vitamin B_6_ deficiency is prevalent in patients with AD, but it is not clear how low vitamin B_6_ status directly influences AD pathogenesis or progression. Patients with AD are more likely to have low plasma PLP concentrations [145]. Combined high vitamin B_6_ and magnesium supplementation was reported to improve verbal communication, non-verbal communication, interpersonal skills, and/or physiological function in children with autism spectrum disorders, but a systematic review concluded that the efficacy was inconclusive [146]. Further studies are expected regarding vitamin B_6_ status, KAT activity, and a KYNA level in patients with dementia.

### 8.4. Indole-3-Pyruvic Acid Precursor and Reactive Oxygen Species

KYNA is also formed at least partly from IPA, which is the transamination product of TRP by the TRP transaminase. It was reported that IPA administration increased 5-HT, 5-hydroxyindole-3-acetic acid, TRP, and KYNA levels in the brain [147]. IPA increases a KYNA level through TRP formation; furthermore, IPA can be converted to KYNA by redox reactions without enzymes. IPA is present in keto or enol tautomer. The latter cleaves the pyrrole ring by reactive oxygen radicals to form KYNA by spontaneous cyclization. IPA tautomerase increases the enol tautomer, favoring a greater formation of KYNA in the presence of free radicals [148].

In addition, KYNA is also produced from l-KYN in the presence of oxidants and peroxidase. KYN donates hydrogen, forming an unstable imino acid, which is the hydrolyzed to 2-oxo acid and ammonia. The 2-oxo acid spontaneously cyclizes to form KYNA [149]. The reaction takes place in the physiological pH ranges in the presence of H_2_O_2._ [150]. d-KYN was observed to produce KYNA with an interaction with hydroxyl radical and peroxynitrite in cerebellum homogenates. In vivo microdialysis studies showed that the KYNA level increases by intracerebellar infusion of l- or d-KYN, peroxynitrite infusion, and intracerebellar infusion of l- or d-KYN after peroxynitrite infusion. KYNA production from d-KYN was not influenced in the presence of a KAT inhibitor, aminooxyacetic acid, compared to one from L-KYN, suggesting that KAT is less responsible for KYNA production from d-KYN [151].

In the presence of peroxynitrite and aminooxyacetic acid, KYNA production from l-KYN decreased by 20%, but no significant change was observed with d-KYN. It suggests a minimal participation of KAT in the persistence of ROS. Furthermore, KYNA productions decreased from both enantiomers by 50% in the presence of an antioxidant, nordihydroguaiaretic acid, suggesting the oxidizing environments that facilitate KYNA production [152]. Both l-KYN and d-KYN are good ROS scavengers and lead to the production of KYNA. Oxidizing environments are in favor of producing KYNA, which may have relevance in brain development and aging and in neurological diseases that show redox environment alteration.

### 8.5. Amino Acid Oxidase and d-Amino Acids

DAAO oxidizes d-amino acids to the corresponding amino acids, producing ammonia and hydrogen peroxide. d-Serine is a physiological agonist at the NMDAR in the brain [153]. d-Enantiomers of amino acids are present at high concentrations in humans and to have biological functions. Derived from microorganisms or l-d racemization, d-amino acids are a pool of l-isomers that are necessary for protein synthesis and antagonists for l-isomers at biological sites. Bacterial pathogens and immune activation may cause an imbalance of d-amino acid concentrations [154].

D-TRP can be usable to promote growth in a TRP-deficient diet, and d-TRP and l-TRP were found to be equally effective in the growth of rats. d-TRP and d-KYN were metabolized slower than their l-enantiomers in rat liver. Small amounts of l-KYN, d-KYN, and KYNA were found converted from d-TRP [152]. KYNA and IPA were excreted from d-TRP or d-KYN-supplied rabbits [155]. d-Formyl-KYN was found to be the intermediate of d-TRP to d-KYN conversion, which was inhibited by l-TRP, and KYNA can be converted from d-KYN by DAAO in kidney homogenates [156]. Thus, KYNA can be produced from a d-TRP enantiomer. The intraperitoneal administration of d-TRP or d-KYN increased plasma KYNA levels in rats, which was inhibited by a DAAO inhibitor, 5-methylpyrazole-3-carboxylic acid [157]. KYNA was found to be produced from d-KYN in human brains, the KYN production being the highest in the cerebellum [158]. Microdialysis studies showed that increase in KYNA levels were observed after the intraperitoneal (i.p.) administration of d- or l-TRP or the infusion of d-KYN in the prefrontal cortex, which was inhibited by the DAAO inhibitor. In vitro studies showed that the KAT inhibitor inhibited KYNA production from d-KYN by 30% and the DAAO inhibitor inhibited it by 70% [152]. I.p. injection of d-TRP increased l-TRP levels in the plasma, forebrain, and cerebellum, confirming d-TRP to l-TRP conversion. KYNA levels were decreased by DAAO inhibitor in cerebellum, suggesting that DAAO takes a main role in KYNA production in cerebellum [159]. d-TRP and d-KYN are normally present in normal conditions by food intake and conversion by gastrointestinal microorganisms [152]. Thus, d-enantiomers influence a level of l-KYNA which may be affected by alteration of the cerebral DAAO activity in inflammation and neurological disorders.

## 9. Conclusions

New AD drugs have been explored allosterically to approach the etiological targets, including Aβ, cholinergic neurotransmission, NMDARs, tau proteins, neurovasculation, inflammation, or virus. More than 190 compounds have been tested, and more than 400 clinical trials are currently taking place. The failed clinical trials have been attributed to the possible heterogenous etiology of AD, which converges though different routes into a common pathological and clinical vignette: neurodegeneration and dementia. Only two classes of drugs have been approved so far for clinical use for the treatment of AD, one of which is the NMDA antagonist, memantine. Memantine is indeed a NMDAR modulator with weak agonistic activity and multiple target sites. Thus, it may be worth exploring novel lead compounds with similar biological activities to that of memantine. On the other hand, the disturbance of TRP metabolism has been observed in the plasma and CSF of patients with dementia, and a branch of TRP metabolism, the KYN pathway, has been found to be closely linked to AD pathogenesis in which multiple positive feedback loops through disturbed KYN metabolites may potentially contribute to the exacerbation of dementia.

One of the KYN metabolites, KYNA, is an endogenous NMDAR inhibitor with multiple targets and actions against neuroexcitotoxicity, inflammation, and ROS. Being an active antioxidant compound on its own right, KYNA triggers neuromodulatory actions through multiple routes including the NMDAR, GPR35, and AhR. In addition, KYNA exhibits excitatory and inhibitory dual actions at AMPA receptors in a dose-dependent manner. It has been observed that levels of neurotoxic KYN metabolites are increased and those of neuroprotective KYNA are decreased in patients with AD, PD, HD, and MDD. Thus, modifying KYNA levels may be a potential approach to normalize TRP metabolism and potentially alleviate positive feedback loops connecting to multiple AD pathogeneses and dementia.

Possible strategies and relevant mechanisms to modify a level of KYNA are reviewed, including the design of BBB-permeable prodrugs metabolized to KYNA upon the entry to brain; the design of highly BBB-permeable KYNA analogues with halogenation, conjugation, and nanotechnology; and KAT enzyme potentiation. Furthermore, another KYNA formation pathway from IPA and KYNA production in the absence of enzymes, and potential roles of d-enantiomers and DAAO are also discussed.

The delivery of KYNA prodrugs, administration of KYNA analogues and vitamin B_6_ supplements, maintenance of adequate d-enantiomer reservoir, and monitoring of DAAO activities of the gastrointestinal microbiota may of benefit to counteract the disturbance of the KYN pathway and thus potentially alleviate the exacerbation of multiple feedback loops of AD pathogenesis and dementia. The design of multitargeting KYNA derivatives in a holistic approach to heterogenous targets of dementia to alleviate positive feedback loops by regulating KYN metabolism may be of great value in the search for novel lead compounds against dementia.

## Figures and Tables

**Table 1:** Studies included for systematic review synthesis, study designs, and risk bias assessment.

Diseases	Study Types	Reference Numbers or Sample Numbers (Disease/Control)	Samples	Risk of Bias
***Neurodegenerative diseases***				
**Alzheimer’s disease [50,51,52,53,54]**				
Guillemin et al., 2005 [51]	case-control study	6/4	brain tissue	High risk
Bonda et al., 2010 [52]	case-control study	12/7	brain tissue	
Gulaj et al., 2010 [53]	case-control study	34/18	serum	
Schwarcz et al., 2013 [54]	case-control study	20/19	serum	
**Parkinson’s disease [55,56,57,58,59]**				
Hartai et al., 2005 [57]	case-control study	19/17	plasma, RBC	High risk
Lewitt et al., 2013 [58]	case-control study	48/57	CSF	
Chang et al., 2018 [59]	case-control study	118/37	plasma	
**Huntington’s disease [60,61,62,63,64]**				
Reynolds and Pearson, 1989 [64]	case-control study	12/11	postmortem brain tissue	High risk
Beal et al., 1992 [62]	case-control study	14–30/25–40	postmortem brain tissue	
Jauch et al., 1995 [63]	case-control study	17/17	postmortem brain tissue	
Stoy et al., 2005 [61]	case-control study	15/11	plasma	
**Vascular Cognitive Dementia**				Unclear
Darlington et al., 2007 [65]	case-control study	50/35	serum	
Yan et al., 2015 [66]	case-control study	28/20,11	serum, CSF	
***Psychiatric disorders***				
**Major depressive disorder**				Low risk
Ogawa et al., 2014 [67]	meta-analysis	10	plasma	
Réus et al., 2015 [68]	systematic review	29	plasma, blood, serum, CSF, urine, brain tissue	
Ogyu et al., 2018 [69]	meta-analysis	22	plasma	
**Bipolar disorder [70,71,72,73,74,75,76,77]**				
Birner et al., 2017 [75]	case-control study	143/101	blood	Unclear
Wang et al., 2018 [76]	meta-analysis	16	CSF	
Arnone et al., 2018 [77]	meta-analysis	5	serum	
**Generalized anxiety disorder**				
Orlikov et al., 1994 [78]	case-control study	16/15	plasma	Unclear
Altmaier et al., 2013 [79]	case-control study	386/116	serum	
**Autism spectrum disorder**				
Lim et al., 2016 [80]	case-control study	15/12	blood	Unclear
Bryn et al., 2017 [81]	case-control study	30/30	serum	

**Table 2:** Systematic synthesis of kynurenine levels in neurodegenerative diseases and psychiatric disorders. ↑: increase; ↓: decrease; ?: unclear or unknown.

Diseases	Neurotoxic Kynurenines	Neuromodulatory Kynurenines
***Neurodegenerative diseases***		
** Alzheimer’s disease**	↑	↓
** Parkinson’s disease**	↑	↓
** Huntington’s disease**	↑	↓
** Vascular cognitive dementia**	↑	↑
***Psychiatric disorders***		
** Major depressive disorder**	↑	↓
** Bipolar disorder**	**?**	**?**
** Generalized anxiety disorder**	↓	**?**
** Autism spectrum disorder**	↑	**?**

**Table 3:** NMDAR modulators approved for clinical use and under clinical trials. Only memantine is approved for clinical use for Alzheimer’s disease (AD). Gavestinel failed to show efficacy against ischemic stroke. AVP-786, AXS-05, B1425809, and DAOI are under clinical trials. NMDAR: N-methyl-D-aspartate glutamatergic receptor, MDD: major depressive disorder.

NMDAR Modulators	Modes	Status	Ref.
Memantine	NMDAR antagonist	Approved for moderate to severe AD	[96]
Gavestinel (GV150,526A)	NMDAR antagonist	No efficacy in ischemic stroke under Phase 3 trials	[105]
AVP-786 (Nuedexta)	NMDAR antagonist	Approved for the treatment of pseudobulbar affect No benefit in three Phase 3 trials against agitation of AD	[106]
AXS-05	NMDAR antagonist Combination drug	Treatment-resistant MDD and agitation in AD	[106]
BI425809	NMDAR agonist	Cognitive impairment of AD and schizophrenia	[107]
DAOI	NMDAR modulator	Cognitive impairment of AD under Phase 2 clinical trials	[108]

**Table 4:** Targets of kynurenic acid. Kynurenic acid (KYNA) has multiple targets including NMDA receptor (NMDAR), inflammatory cells, G protein-coupled receptor 35 (GPR35), aryl hydrocarbon receptor (AhR), reactive oxygen species (ROS), and kynureninase A. Its actions include anti-excitotoxic, anti-inflammatory, antioxidant, and immunomodulatory activities. AMPA: α-amino-3-hydroxy-5-methyl-4-isoxazolepropionic acid.

Targets	Ref.
**1. NMDA Receptor (NMDAR)** -Inhibits strychnine-insensitive glycine-binding site of the NR1 subunit of NMDAR -Inhibits glutamate-binding site to the NR2 subunit of NMDAR -Inhibits the presynaptic α7 nicotinic Ach receptors (controversial) -Stimulates/inhibits at the AMPA receptor in a dose-dependent manner	[45] [109] [110] [11] [112]
**2. Inflammatory Cells** -Reduces TNF expression and secretion -Decreases high-mobility group box 1 protein secretion -Inhibits α-defensin human neutrophil peptides 1–3 secretion -Reduces IL-4 release in invariant natural killer-like T cell -Reduces lipopolysaccharide-induced IL-23 expression -Inhibits Th17 cell differentiation	[89]
**3. G Protein-Coupled Receptor 35 (GPR35)** -Activates GPR35 signaling -Reduce pro-inflammatory cytokines release -Analgesic effects in inflammatory models -N-type calcium channel inhibition	[113] [114]
**4. Aryl Hydrocarbon Receptor (AhR)** -Regulates cellular differentiation, cellular adhesion and migration, and immune response -Induces adaptive immunity by modulating T-cell differentiation and function	[115] [116]
**5. Reactive Oxygen Species (ROS)** -Abolishes ROS production produced by FeSO_4_	[41] [117]
**6. Kynureniase A** -Stimulates the production of AA, which suppresses pro-inflammatory IFN-γ, T and B lymphocyte cell proliferation, and Th1 cell activity, while it increases anti-inflammatory cytokine IL-10	[118] [119] [120]

**Figure A1:**
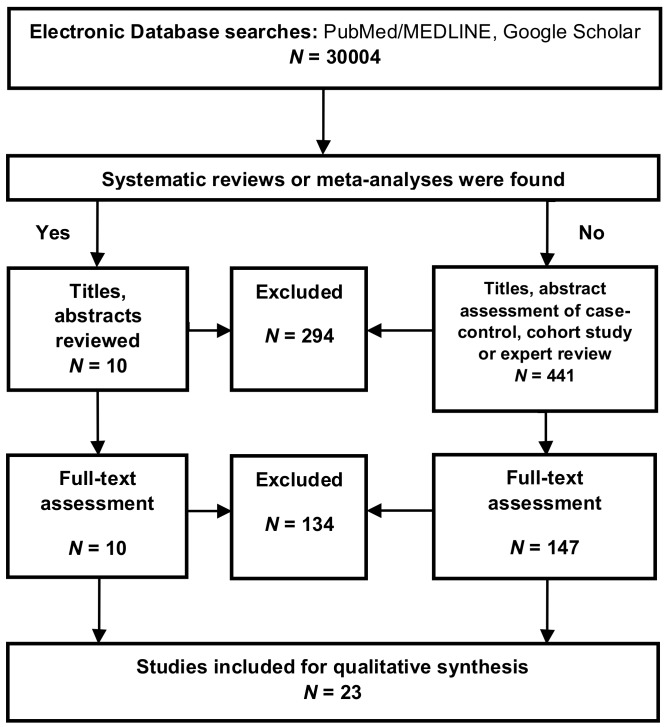
Flow diagram of qualitative synthesis adopted from Preferred Reporting Items for Systematic Reviews and Meta-Analyses (PRISMA).

## Appendix A

In Section 1, Section 2, Section 5, Section 6, Section 7 and Section 8, a literature search was employed in PubMed/MEDLINE and Google Scholar, which ranged from database inception to December 2019, and the relevant keywords including their synonyms and combinations were used search terms such as “dementia”, “alzheimer disease”, “kynurenine”, “kynurenic acid”, “tryptophan”, “indoleamine 2,3 dioxygenase”, “tryptophan 2,3-dioxygenase”, “kynurenine aminotransferase”, “kynurenine 3 monooxygenase”, “antagonists, glutamate”, “NMDA receptors”, “memantine”, “clinical trial”, “drug design”, etc. The search filters include “English”, “review”, “systematic review”, “meta-anlysis”, etc. In Section 4, a systematic review methodology was adopted from Preferred Reporting Items for Systematic Reviews and Meta-Analyses (PRISMA) [160]. A literature search was employed in PubMed/MEDLINE and Google Scholar. M.T. searched and assessed eligibility, and further extracted data (Figure A1).

### Appendix A.1. Inclusion Criteria

Articles included in the review article were selected according to the following criteria: (1) articles published as an original article; (2) articles providing sufficient information of diseased populations healthy controls; (3) articles written in English; and (4) articles retrievable online. Studies of patients under medications were included.

### Appendix A.2. ExInclusion Criteria

Articles excluded were the following: (1) articles of no original data; (2) articles of animal studies; and (3) articles of no controls except for longitudinal cohort study.

### Appendix A.3. Selection Process

The search ranged from database inception to December 2019, and the relevant keywords including their synonyms and combinations were used as search terms of “neurodegenerative diseases”, “psychiatry”, “dementia”, “alzheimer disease”, “parkinson disease”, “huntington disease”, “vascular dementia”, “disorder, major depressive”, “bipolar disorder”, “anxiety disorder”, “autism spectrum disorder”, “tryptophan”, “kynurenine”, “kynurenic acid”, etc. After excluding duplicates and reviewing titles and abstracts, the full texts of articles were assessed. The search priority was given in the following order: meta-analysis, systematic review, case-control study, cohort study, and review. If no meta-analysis or systematic review was found in the search, the full-text articles of case-control studies, cohort studies, and reviews were assessed for eligibility.

### Appendix A.4. Data Extraction

Data from eligible articles were extracted into a table for qualitative analysis and critical assessment. Data collected from each study include year of publication, study design, the number of articles or diseased and healthy populations, and sample types.

### Appendix A.5. Assessment of the Methodological Quality

The methodological quality was assessed for each neuropsychiatric disease according to the presence and number of study design.

### Appendix A.6. Risk of Bias Assessment

Risk of bias assessment was adopted and conducted from the Cochrane Handbook for Systematic Reviews of Interventions [161]. The criteria of diagnosis and levels of neurotoxic KYNs and modulatory KYNs were assessed according to availability of studies, study types, and study results, and evidence levels were judged into high risk, low risk, or unclear (Table A1).

**Table A1:** Neurotoxic and modulatory KYN levels were assessed according to the criteria of availability of meta-analysis or systematic review, study types, and with or without conflicting results, in order to judge evidence levels of high risk, low risk, or unclear.

Risk of Bias	Criteria
High risk	No meta-analysis or systematic review, less than five case-control and/or cohort studies, or presence of only expert review
Low risk	Presence of at least one meta-analysis or systematic review, without conflicting results
Unclear	Presence of only case-control study or cohort study, meta-analysis with conflicting results, or case-control studies with conflicting results
